# Preventive Role of Wire-Guided Cannulation to Reduce Hyperamylasemia and Pancreatitis Following Endoscopic Retrograde Cholangiopancreatography

**DOI:** 10.1155/2012/821376

**Published:** 2012-07-15

**Authors:** Amir Houshang Mohammad Alizadeh, Esmaeil Shamsi Afzali, Siavash zafar Doagoo, Mirhadi Mousavi, Dariush Mirsattari, Anahita Shahnazi, Mohammad Reza Zali

**Affiliations:** Research Center for Gastroenterology and Liver Diseases, Shahid Beheshti University of Medical Sciences, Taleghani Hospital, P.O. Box 19835-178, Tehran, Iran

## Abstract

*Background and Study Aims.* The usefulness of wire-guided cannulation for avoiding hyperamylasemia and pancreatitis following endoscopic retrograde cholangiopancreatography (ERCP) is conflicting, and therefore we designed this study to determine whether wire-guided cannulation reduces the rate of post-ERCP hyperamylasemia and pancreatitis and compare its efficacy to conventional method. *Patients and Methods.* Seven hundred and forty-eight patients with hepatobiliary diseases consecutively underwent diagnostic or therapeutic ERCP at the unit of Taleghani referral hospital in Tehran. Among them, 546 patients were eligible for wire-guided cannulation and underwent this procedure and others underwent sphincterotome biliary cannulation using contrast injection as the conventional method. *Results.* Patients in the two groups were comparable in terms of gender and age. Successful biliary cannulation was achieved similary in the guidewire and conventional group (89.2% versus 86.4%) that in 5.4% and 14.1% of them it was difficultly performed, respectively (*P* = 0.003). The main pancreatic duct was more visualized in 99.0% of patients in conventional group in comparison with 79.0% in another group (*P* < 0.001). Multivariate regression analysis showed that wire-guided cannulation had a protective role for post-ERCP hyperamylasemia (OR: 0.336, 95% CI: 0.181–0.623, *P* < 0.001). However, there were no significant differences between the two groups in rates of other procedure-related complications, such as, pancreatitis, bleeding, and perforation. *Conclusion.* The use of guidewire cannulation in comparison with conventional method can be accompanied with lower post-ERCP hyperamylasemia, and therefore selection of this cannulation technique especially in high-risk group is recommended.

## 1. Introduction 

Insertion of a guidewire is one of the most applicable techniques with high success rate and few probable complications after selective cannulation of common bile duct. More efficacy of this procedure has been found in comparison with conventional methods of cannulation with contrast injection in some recent studies [1]. It has been suggested that accessing the bile duct with guidewire can lead to reducing traumatic injury of pancreatic duct and papilla and avoiding hydrostatic pressure associated with contrast injection [2, 3]. In addition, advertent injection of contrast agents into the pancreatic duct has been confirmed to play a pivotal role in the development of some complications after endoscopic retrograde cholangiopancreatography (ERCP), such as, pancreatitis and the degree of pancreatic duct opacification has been associated with this complication [4]. However, some other trials could not confirm the improvement of biliary cannulation success rate by using the guide-wire technique and suggested that the role of skill and experience of the proceduralist was more important than whether guidewire or contrast to achieve biliary cannulation and minimize post-ERCP pancreatitis [5].

According to considerable reported incidence of post-ERCP pancreatitis as a common complication, it seems that the findings of studies regarding the usefulness of wire-guided cannulation for avoiding this complication are conflicting and a few studies are published comparing guidewire with contrast-assisted biliary cannulation. The present study was performed to determine whether wire-guided cannulation reduces the rate of post-ERCP hyperamylasemia and pancreatitis and compare its efficacy to conventional method. 

## 2. Patients and Methods 

### 2.1. Study Population

This retrospective study was done on patients' records registered in a gastroenterology and hepatology ward of a referral hospital in Tehran, Iran. A total of 748 patients with hepatobiliary diseases and candidate for diagnostic or therapeutic ERCP and referred to Taleghani referral hospital between 2006 and 2011 were underwent ERCP procedure. The study was approved by the ethics committee of Shahid Beheshti University of Medical Sciences. Among them, 546 patients were eligible for wire-guided cannulation and underwent this procedure and others underwent sphincterotome biliary cannulation using contrast injection as the conventional method. After introducing guide wire cannulation in this center, it was chosen at the endoscopists' judgment based of patients' characteristics and situation. Our center is an academic center training fellowship of gastroenterology and hepatology. ERCP was performed first by fellowship and continued the by an expert endoscopist. Patients with these criteria were ineligible: age below 15 years, acute illness, such as, hypotension, hypoxia, oxygen saturation less than 95% on supplemental oxygen, and hemodynamic instability. Furthermore, patients with surgically altered anatomy (Billroth II or Roux-en-Yanastomosis) were also excluded as cannulation technique is then fundamentally different from that in normal anatomy [5]. All patients signed research study informed consent before ERCP. Patients' baseline data were collected and recorded by interviewing in the day of admission to hospital included: demographic characteristics, medical history, and clinical presentations. Laboratory parameters were also measured in the day of admission that consisted of cell blood count and liver function tests. The indications for ERCP were also recorded.

### 2.2. Cannulation Protocol and Outcome

Eligible patients underwent ERCP for suspected and diagnosed pancreatobiliary disease and on the basis of generally accepted diagnostic indications for ERCP [6]. Procedure was performed under conscious sedation with midazolam and meperidine and by a staff gastroenterologist. Cannulation was performed in each group on the basis of techniques as previously described [1].

Successful cannulation was defined as free and deep instrumentation of the biliary tree, and a cannulation attempt was defined as sustained contact with the cannulating device and the papilla for at least five seconds [5]. Difficult biliary cannulation was also related to the failure of biliary access despite ten minutes of attempted biliary cannulation or more than five attempted unintentional pancreatic cannulations [7]. Serum amylase was also measured 30 minutes before and 3 hours after cannulations. Post-ERCP hyperamylasemia was defined as an elevation of the serum amylase level above the upper normal limit (160 IU/L). Furthermore, post-ERCP pancreatitis was defined as new or worsened abdominal pain with elevation of serum amylase at least three times above the upper normal limits for 24 hours after a procedure, requiring hospitalization or prolongation of a planned admission [8]. Other studied complications included jejunal or duodenal perforation, local bleeding, and cholangitis.

### 2.3. Statistical Analysis

Results were reported as mean ± standard deviation (SD) for quantitative variables and percentages for categorical variables. The groups were compared using the Student's *t*-test for continuous variables and the chi-square test for categorical variables. We used multivariate logistic regression analysis to investigate the potential confounding effects of patients' characteristics and clinical data on the relationship between cannulation technique and procedure outcome. Odds Ratio (OR) and 95% Confidence Interval for OR were also calculated. *P* values of 0.05 or less was considered statistically significant. All the statistical analyses were performed using SPSS version 13.0 (SPSS Inc., Chicago, IL, USA).

## 3. Results 

Patients in the two groups were comparable in terms of gender and age. Male to female ratio in guide-wire and conventional groups were 1.0 and 0.9, respectively. Also, mean age of patients in the two groups was 58.5 ± 16.9 and 55.7 ± 17.6 years, respectively. Regarding medical history, those who underwent conventional methods of cannulation with contrast injection had lower history of biliary stones and previous ERCP compared to guide-wire group. However, other risk factors, such as, diabetes mellitus, hypertension, coronary artery disease, smoking, and opium addiction were comparable in the two groups (Table 1). Successful biliary cannulation was achieved in 89.2% of the patients in guide-wire group and in 86.4% of patients in the conventional group (*P* > 0.05) that in 3.0% and 10.3% of them was difficult to be performed, respectively (*P* < 0.05). The main respective indications for ERCP in guidewire and conventional groups (Table 2) were as follows: choledocholithiasis (77.8% versus 67.3%), cholangiocarcinoma (5.7% versus 6.4%), and pancreatic head cancer (6.0% versus 10.4%). The main pancreatic duct was visualized in 99.0% of patients in conventional group in comparison with 79.0% in guide-wire group that was significantly found higher in the first group group (*P* < 0.001).

A post-ERCP hyperamylasemia was found in 15.6% of all studied patients. This complication was significantly lower in the guide-wire group (1.4%) compared to the conventional group (20.8%).

**Figure 1:**
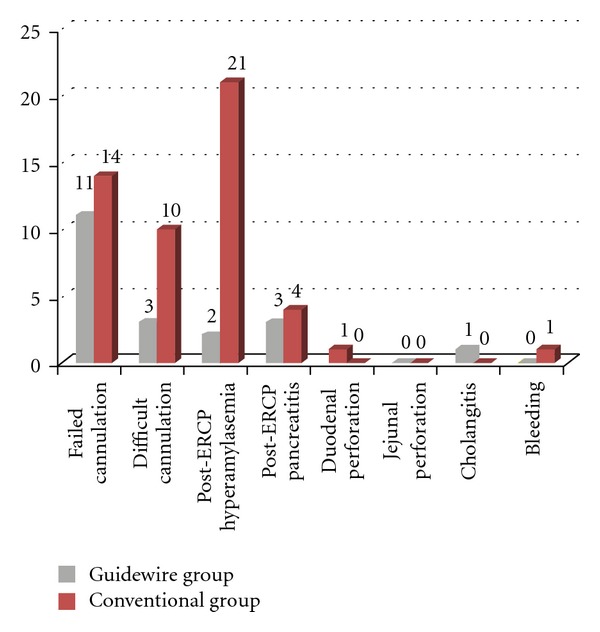
ERCP outcome in guidewire and conventional therapy groups: post-ERCP hyperamylasemia was less occurred following wire-guided cannulation in comparison with conventional techniques (*P* < 0.05).

Multivariate regression analysis also showed that wire-guided cannulation had a protective role for post-ERCP hyperamylasemia (OR: 0.336, 95% CI: 0.181–0.623, *P* < 0.001). However, there were no significant differences between guidewire and conventional group in term of other procedure-related complication, such as, pancreatitis (2.8% versus 4.5%), bleeding (0.0% versus 1.0%), perforation (0.9% versus 0.0%), and cholangitis (0.7% versus 0.0%) (Figure 1).

## 4. Discussion

Post-ERCP pancreatitis has been suggested one of the most common and serious complications that can result in substantial considerable morbidity and even mortality [8–11]. In some studies, pancreatitis was noted as the most common complication of ERCP [12]. The incidence of this complication had a wide range according to recent large prospective studies from 1% to 19.5% [13–17]. In our study, overall incidence rate of pancreatitis was estimated as 3.3%. Wide rate of post-ERCP pancreatitis, incidence in different studies can be related to the threshold for serum amylase level to define post-ERCP pancreatitis, and thus this factor potentially leads to underestimate or overestimate the incidence of this complication. In addition, this rate can be influenced by patient susceptibility, the endoscopist skills and experiences and also the thoroughness of followup [18, 19]. More over, despite using of defined criteria for diagnosis of acute pancreatitis in most patients, the criteria cannot be always accurate in patients following ERCP so that many patients with post-ERCP pancreatitis have some of these criteria in the absence of acute pancreatitis, pain, and an elevation of amylase or lipase [20]. 

Several patient- and procedure-related factors have been known effective on the occurrence of hyperamylasemia and post-ERCP pancreatitis, such as, younger age, female sex, previous ERCP-induced pancreatitis, and sphincter of Oddi dysfunction [16]. Cheng et al. in a multicenter study indicated the incidence of pancreatitis development in 15.1% of patients undergoing ERCP. In their survey, significant risk factors for this event were minor papilla sphincterotomy, suspected sphincter of Oddi dysfunction, history of post-ERCP pancreatitis, age < 60 yr, ≥2 contrast injections into the pancreatic duct, and trainee involvement [21]. 

The influence of cannulation technique especially contrast injections into the pancreatic duct is questioned. Our study showed that the use of guidewire for cannulation effectively reduced the occurrence of post-ERCP hyperamylasemia, however, had no significantly effect on the occurrence of pancreatitis. Furthermore, difficult cannulation and pancreatic duct injection were observed less frequent in guide-wire method, and this difference can be the main cause of lower hyperamylasemia following this technique. Similar to our study, in Lee et al. study, biliary cannulation was achieved more successful in the wire-guided cannulation than conventional method with contrast injection. Also, they found that a hyperamylasemia at 24 hours after ERCP was lower in the patients who cannulated by guide-wire than another group. However, contrary to our study, guidewire cannulation had a main predictor for post-ERCP pancreatitis [1]. In the study by Lella et al, none of patient was cannulated with guidewire had pancreatitis, and this complication was found only in 4.1% of the control group [3]. In another study by Artifon et al. the use of guidewire technique for bile duct cannulation led to lower rate of post-ERCP pancreatitis than conventional method (8.6% versus 16.6%) [2]. Some probable causes of post-ERCP prevention of hyperamylasemia or pancreatitis following guidewire cannulation include preventing unintentional submucosal injection of contrast media into the main pancreatic duct or the papilla [3] and facilitating cannulation by limiting papillary trauma and the need to precut sphincterotomies [2]. 

Besides, in some other studies, post-ERCP pancreatitis occurred or more in guidewire cannulation than conventional method or the incidence of this event was similar between the two techniques. In Bailey et al. study, post-ERCP pancreatitis occurred similarly in cannulation with a guide-wire and conventional contrast-assisted cannulation with the rates of 7.9% and 6.2%, respectively, and overall rate of 7.0%. Furthermore, in their trial, although the guidewire technique improved the success rate for biliary cannulation during ERCP, it could not prevent the incidence of post-ERCP pancreatitis compared to the conventional contrast technique [5]. In another study by Tsuyuguchi et al. difficult cannulation technique and stenting were not significant risk factors for post-ERCP pancreatitis [13]. It seems that their adverse findings could be due to difficult wire passages that resulted in injury to the papilla or the use of this technique as rescue method in patients with failed conventional cannulation. Totally, it seems that an evaluation and development safer cannulation technique that can minimize the number of injections into the pancreatic duct and enhances selective cannulation is an important role of endoscopists. Also, routine use of pancreatic duct stent placement especially in high-risk patient population especially in those with suspected sphincter of oddi dysfunction is recommended. 

In summary, the use of guidewire cannulation in comparison with conventional method can be accompanied with lower post-ERCP hyperamylasemia, and therefore selection of this cannulation technique especially in high-risk group is recommended.

## Figures and Tables

**Table 1:** Baseline characteristics and medical history in guidewire and conventional therapy groups.

Characteristics	Guidewire group (*n* = 546)	Conventional group (*n* = 202)	*P* value
Female gender	270 (49.5)	105 (52.0)	0.724
Age (Years)	58.5 ± 16.9	55.7 ± 17.6	0.065
Medical history			
Diabetes mellitus	63 (11.5)	20 (9.9)	0.570
Hypertension	107 (19.6)	31 (15.3)	0.266
Coronary artery disease	51 (9.3)	10 (5.0)	0.070
Cigarette smoking	65 (11.9)	26 (12.9)	0.751
Alcohol using	16 (2.9)	5 (2.5)	0.745
Opium addiction	29 (5.3)	11 (5.4)	0.945
Cholecystectomy	198 (36.3)	71 (35.1)	0.846
Previous ERCP	63 (11.5)	4 (2.0)	<0.001
Biliary stone	74 (13.6)	1 (0.5)	<0.001
Cirrhosis	8 (1.5)	3 (1.5)	0.999

Data are presented as mean ± SD or number (percentage).

**Table 2:** Indications for ERCP in guidewire and conventional therapy groups.

Characteristics	Guidewire group (*n* = 546)	Conventional group (*n* = 202)
Choledocholithiasis	425 (77.8)	136 (67.3)
Cholangiocarcinoma	31 (5.7)	13 (6.4)
Pancreatic head cancer	33 (6.0)	21 (10.4)
Suspected sphincter of Oddi dysfunction	138 (25.3)	57 (28.2)
Primary sclerosing cholangitis	12 (2.2)	15 (7.4)
Others	43 (7.9)	15 (7.4)

Data are presented as number (percentage).
